# Prognostic Role of CA-125 Elimination Rate Constant (KELIM) in Patients with Advanced Epithelial Ovarian Cancer Who Received PARP Inhibitors

**DOI:** 10.3390/cancers16132339

**Published:** 2024-06-26

**Authors:** Ji Hyun Kim, Eun Taeg Kim, Se Ik Kim, Eun Young Park, Min Young Park, Sang-Yoon Park, Myong Cheol Lim

**Affiliations:** 1 Center for Gynecologic Cancer, National Cancer Center, Goyang 10408, Republic of Korea; gynlittle@gmail.com (J.H.K.); parksang@ncc.re.kr (S.-Y.P.); 2 Department of Obstetrics and Gynecology, Kosin University College of Medicine, Pusan 49241, Republic of Korea; dikei03@naver.com; 3 Department of Obstetrics and Gynecology, Seoul National University College of Medicine, Seoul 03080, Republic of Korea; seikky@naver.com (S.I.K.);; 4 Biostatistics Collaboration Team, Research Core Center, National Cancer Center, Goyang 10408, Republic of Korea; 13140@ncc.re.kr; 5 Rare & Paediatric Cancer Branch and Immuno-Oncology Branch, Research Institute, National Cancer Center, Goyang 10408, Republic of Korea; 6 Department of Cancer Control and Policy, Graduate School of Cancer Science and Policy, National Cancer Center, Goyang 10408, Republic of Korea

**Keywords:** ovarian cancer, PARP inhibitors, prognosis, progression-free survival, CA-125

## Abstract

**Simple Summary:**

Prior research has identified various prognostic markers in epithelial ovarian cancer (EOC), including BRCA mutation status and a response to platinum-based chemotherapy, to predict outcomes in patients undergoing PARP inhibitor maintenance therapy. The role of CA-125 elimination rate constant K (KELIM), although recognized as a prognostic indicator, has not been fully investigated. This study underscores the prognostic significance of KELIM, revealing that a favorable KELIM score significantly correlates with better PFS in patients treated with primary cytoreductive surgery (PCS) followed by PARP inhibitor therapy. It also shows that KELIM’s predictive value varies with the timing of surgery, extending a different view of its utility in real-world practice. KELIM could be integrated into clinical decision-making processes, potentially informing future clinical guidelines and research into optimal treatment strategies for targeted use of PARP inhibitors in advanced EOC patients.

**Abstract:**

Background: This multicenter retrospective study aimed to investigate the prognostic value of the CA-125 elimination rate constant K (KELIM) in EOC patients who received platinum-based chemotherapy followed by PARP inhibitors, in either upfront or interval treatment settings. Methods: Between July 2019 and November 2022, we identified stage III–IV EOC patients who underwent primary or interval cytoreductive surgery and received olaparib or niraparib. Individual KELIM values were assessed based on validated kinetics and classified into favorable and unfavorable cohorts. Results: In a study of 252 patients undergoing frontline maintenance therapy with olaparib or niraparib, favorable KELIM (≥1) scores were associated with a higher PFS benefit in the primary cytoreductive surgery (PCS) cohort (hazard ratio (HR) for disease progression or death 3.51, 95% confidence interval (CI); 1.37–8.97, *p* = 0.009). Additionally, within the interval cytoreductive surgery (ICS) cohort, a favorable KELIM score (≥1) significantly increased the likelihood of achieving complete resection following cytoreductive surgery, with 59.4% in the favorable KELIM group compared to 37.8% in those with unfavorable KELIM. Conclusions: A favorable KELIM score was associated with improved PFS in patients with advanced EOC undergoing PCS. Furthermore, in the ICS cohort, a favorable KELIM score increased the probability of complete cytoreduction.

## 1. Introduction

Epithelial ovarian cancer (EOC) is a major contributor to cancer-related mortality in gynecological malignancies, with the United States expected to report approximately 19,680 new cases and 12,740 deaths in 2024 [1,2]. Platinum-based chemotherapy followed by maintenance therapy with poly (adenosine diphosphate-ribose) polymerase (PARP) inhibitors with or without bevacizumab has emerged as the standard frontline treatment regimen for women diagnosed with advanced EOC, showing substantial benefits in improving progression-free survival (PFS) and overall survival (OS) rates in the overall population [3,4,5,6,7].

With advancements in treatment modalities, a pressing need remains for reliable prognostic markers to guide which patients truly benefit from PARP inhibitors as maintenance therapies. Several clinical characteristics have been demonstrated to be favorable prognostic factors, including BRCA mutation status, surgical outcomes after cytoreductive surgery (CRS), disease stage, and timing of cytoreductive surgery (upfront or interval surgery) [8]. Notably, the presence of homologous recombination-mediated DNA repair gene mutations, including *BRCA1/2*, has been shown to predict sensitivity to both platinum agents and PARP inhibitors [9,10]. This overlap in molecular characteristics and resistance mechanisms of platinum sensitivity indicates the potential effectiveness of PARP inhibitors [11,12].

The CA-125 elimination rate constant K (KELIM) has recently been introduced as a potential prognostic indicator of platinum sensitivity [13]. KELIM, a kinetic parameter that is calculated from CA-125 measurements taken during the first 100 days of chemotherapy, is a reliable and reproducible indicator of tumor-intrinsic chemosensitivity in the frontline or recurrent setting [13,14,15,16]. However, there is currently no study evaluating its prognostic value specifically in the context of PARP inhibitor maintenance therapy following platinum-based chemotherapy in patients with advanced EOC using real-world data.

The aim of this multicenter retrospective cohort study was to investigate the prognostic value of KELIM in predicting the survival outcomes of women with newly diagnosed advanced epithelial ovarian cancer who received PARP inhibitors as a frontline maintenance treatment after responding to platinum-based chemotherapy.

## 2. Materials and Methods

### 2.1. Study Design

This study is a multicenter cohort study that retrospectively collected data from three Korean institutions as follows: the National Cancer Center, Seoul National University Hospital, and Kosin University Hospital. This study was approved by the Institutional Review Boards of the collaborating institutions and conducted in accordance with the Declaration of Helsinki (National Cancer Center: NCC2023-0079, Seoul National University Hospital: H-2108-169-1248, Kosin University Hospital: KUGH 2023-03-008). The requirement for informed consent was waived.

### 2.2. Patients

Patients were eligible if they (1) completed CRS followed by platinum-based chemotherapy as frontline treatment; (2) had complete or partial response to platinum-based chemotherapy; (3) had histologically confirmed advanced (International Federation of Gynecology and Obstetrics (FIGO) stage III or IV) epithelial ovarian cancer, fallopian cancer, or primary peritoneal cancer between 1 July 2019, and 31 November 2022; and (4) were treated with either olaparib or niraparib as maintenance treatment. We excluded patients who (1) received bevacizumab as frontline treatment, (2) had early stage (FIGO I or II) epithelial ovarian cancer, and (3) had insufficient CA-125 surveillance, or were lost to follow-up during maintenance treatment. The dosage of olaparib was 300 mg tablets twice daily. For niraparib, dosages were 300 mg capsules once daily, or 200 mg when patients had a body weight of less than 77 kg or a platelet count of less than 150,000/µL at the completion of platinum-based chemotherapy. Olaparib was used for up to 2 years after initiation until disease progression, death, or unacceptable toxicity, and niraparib was used until disease progression, death, or unacceptable toxicity.

During maintenance treatment, patients were visited monthly or trimonthly for prescriptions, assessment of hematologic or nonhematologic toxicities, and disease assessment. Disease assessment was performed using CT or PET-CT every 3 months. The date and reason for discontinuation, dose reduction, and interruption of PARP inhibitors were also recorded. The following data were collected for analysis: age, three CA-125 levels during the first 100 days of adjuvant chemotherapy after primary CRS or neoadjuvant chemotherapy before interval CRS, histology, FIGO stage, germline or somatic BRCA mutation status, postoperative residual disease, and response to adjuvant chemotherapy.

### 2.3. Outcome Measures

The primary outcome was the prognostic value of KELIM for PFS, defined as the time interval from completion of platinum-based chemotherapy to the time of disease progression on radiologic assessment according to the Response Evaluation Criteria in Solid Tumors (RECIST) version 1.1, or death from any cause. The KELIM score was calculated according to modeled CA-125 KELIM™, which is assessable online, and kinetics were differently used according to treatment setting (upfront (https://www.biomarker-kinetics.org/CA-125) or interval (https://www.biomarker-kinetics.org/CA-125-neo)).

The secondary outcomes were the likelihood of complete CRS without macroscopic residual disease, according to KELIM (favorable or unfavorable), and adverse events, graded using the Common Terminology Criteria for Adverse Events (CTCAE) version 5.0.

### 2.4. Statistical Analysis

Outcome measures were evaluated separately in the upfront and interval settings to account for different prognoses. Patients’ clinicopathological characteristics were summarized as frequencies with percentages for categorical variables and as a median and interquartile range for continuous variables. The chi-square test or Fisher’s exact test was used to compare the favorable and unfavorable KELIM cohorts for categorical variables, while Student’s t-test or the Wilcoxon rank-sum test was used for continuous variables, as appropriate.

Survival analysis was performed using the Kaplan–Meier method and the Cox proportional hazards model. Kaplan–Meier curves with median survival time are presented, and survival curves between the groups were compared using the log-rank test. The study cohort was divided using a prespecified cutoff of KELIM (1.0). Additionally, we used the Contal and O’Quigley method to calculate the cutoff level as the value that maximizes the Q statistics for each threshold of the cutoff value based on the log-rank test statistics, suggesting an optimal cutoff [17].

To explore factors associated with PFS, the cox proportional hazards model was analyzed for the KELIM score, postoperative residual disease, radiological response after platinum-based chemotherapy, BRCA mutation status, and types of PARP inhibitors. Variables with univariable *p* < 0.2 were included in the multivariable model, and the final model was determined by the backward selection method with an elimination criterion of *p* > 0.05. The results were presented as a hazard ratio with a 95% confidence interval and statistical significance was set at a two-sided *p*-value < 0.05. All statistical analyses were performed using the R project software (version 4.1.1; R Foundation for Statistical Computing, Vienna, Austria) and SAS (version 9.4; SAS Institute Inc., Cary, NC, USA).

## 3. Results

### 3.1. Population

Between July 2019 and November 2022, 252 patients with advanced EOC received either olaparib or niraparib as a frontline maintenance treatment. Of the 252 patients who had at least three CA-125 measurements during the initial three cycles of chemotherapy, 151 (59.9%) underwent primary cytoreductive surgery (PCS) and 101 (40.1%) underwent interval cytoreductive surgery (ICS) after neoadjuvant chemotherapy (Table 1). The PCS cohort included 43 (28.5%) patients with a favorable KELIM score (≥1) and 108 (71.5%) patients with an unfavorable KELIM score (<1). In the ICS cohort, 64 patients (63.4%) had a favorable KELIM score, whereas 37 (36.6%) had an unfavorable score. In the favorable KELIM group, the median KELIM was 1.2 in the PCS cohort and 1.4 in the ICS cohort. In the unfavorable group, the median KELIM was 0.7 in the PCS cohort and 0.8 in the ICS cohort.

In the PCS cohort, baseline characteristics including age at diagnosis, histologic type, FIGO stage, *BRCA1/2* mutation status, treatment type, residual disease, number of cycles of platinum-based chemotherapy, and response to platinum-based chemotherapy were well balanced between the two KELIM score groups (Table 1). In the ICS cohort, the proportion of patients with complete response to platinum-based chemotherapy (*p* = 0.049) and complete resection rates (*p* = 0.037) were higher in the favorable KELIM group.

### 3.2. Association between KELIM and PFS

**Figure 1:**
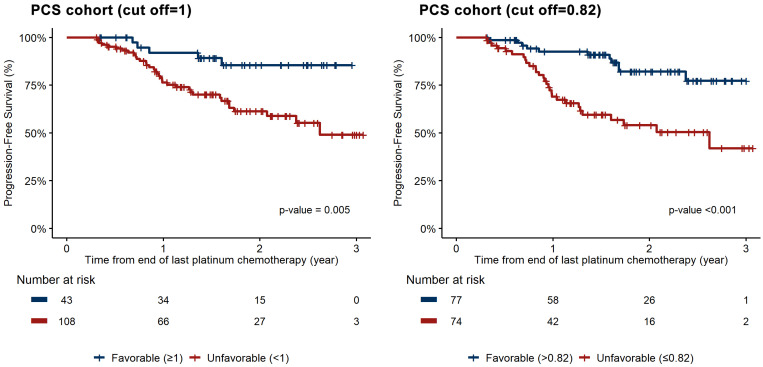
Progression-free survival according to KELIM (favorable or unfavorable) in the cohort of patients who underwent primary cytoreductive surgery: (**left**) cutoff of 1.0; (**right**) cutoff of 0.82.

In the PCS cohort, KELIM status using a prespecified cutoff of 1.0 emerged as a significant prognostic variable in both univariate (hazard ratio for disease progression or death; 3.51, 95% CI; 1.37–8.97, *p* = 0.009) and multivariate survival analyses (HR, 3.03; 95% CI, 1.18–7.76; *p* = 0.021). Utilizing the Contal and O’Quigley method, a suggested cutoff of 0.82 further supported these findings. Applying this cutoff yielded consistent results, showing significance in both univariate (HR, 3.60; 95% CI, 1.79–7.23; *p* < 0.001) and multivariate survival analyses (HR, 3.64; 95% CI, 1.79–7.4; *p* < 0.001) (Table 2). Figure 1 demonstrates the Kaplan–Meier curves for PFS according to KELIM status, showing a difference in PFS between favorable and unfavorable scores (*p* < 0.001).

In addition, germline *BRCA1/2* mutations (pathogenic or likely pathogenic variants) were identified as prognostic covariates in univariate (HR, 4.05; 95% CI, 1.85–8.86; *p* = 0.001) and multivariate survival analyses (HR, 4.02; 95% CI, 1.84–8.78; *p* = 0.001), whereas postoperative residual disease (*p* = 0.099) and radiological response to platinum-based chemotherapy (*p* = 0.688) did not show statistical difference (Table 2).

**Figure 2:**
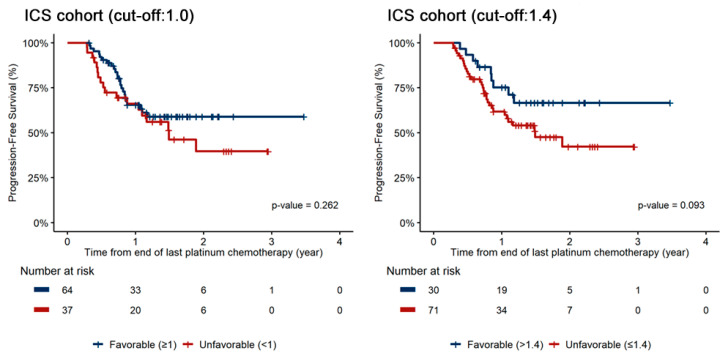
Progression-free survival according to KELIM (favorable or unfavorable) in the cohort of patients who underwent interval cytoreductive surgery after neoadjuvant chemotherapy: (**left**) cutoff of 1.0; (**right**) cutoff of 1.4.

In the ICS cohort, KELIM status was not significantly associated with PFS in univariate analysis (*p* = 0.266). Similarly, in the multivariate analysis adjusted for potential confounders, the KELIM status was not a prognostic covariable (*p* = 0.665) (Table 2). The Kaplan–Meier curves using a KELIM score cutoff of 1.0 in the ICS cohort did not demonstrate a statistically significant difference in PFS (*p* = 0.262). Based on a KELIM score cutoff of 1.4, there was also no significant difference in PFS between patients in the favorable and unfavorable groups (*p* = 0.093) (Figure 2).

Multivariate analysis for PFS showed a poor prognosis for partial response compared to complete response after platinum-based chemotherapy (HR, 4.38; 95% CI, 1.57–12.23; *p* = 0.005) and wild-type or VUS germline *BRCA1/2* mutations compared to PV or LPV *BRCA1/2* mutations (HR, 3.66; 95% CI, 1.80–7.45; *p* < 0.001) (Table 2). In the univariable analysis, postoperative residual disease was associated with worse PFS (HR, 1.98; 95% CI, 1.05–3.75; *p* = 0.035), whereas there was no statistical significance in multivariable analysis after adjustment for clinical factors.

Table S1 shows the patient characteristics in the niraparib and olaparib subgroups. In the multivariable analysis for PFS, the niraparib group showed that a KELIM cutoff of 1.0 was not statistically significant (*p* = 0.207); however, when using the cutoff of 0.82 found by the Contal & O’Quigley method, KELIM became statistically significant (*p* = 0.013) (Table S2). In the olaparib group, KELIM was statistically significant, with the cutoff found by the Contal & O’Quigley method also being 1.0, consistent with the established cutoff (*p* = 0.021) (Table S3).

### 3.3. Safety

In the PCS cohort, the incidence of anemia, thrombocytopenia, and neutropenia did not differ significantly between the patients with favorable and unfavorable KELIM scores. However, nausea was significantly more common in patients with unfavorable KELIM scores (42.6%) than in those with favorable KELIM scores (23.3%, *p* = 0.026). The rates of vomiting, fatigue, abdominal pain, diarrhea, headache, and dose modifications due to treatment-emergent adverse events (TEAE) were similar between the two KELIM groups, with no significant differences in dose reductions, interruptions, or discontinuations due to TEAEs (Table S4).

In the ICS cohort, similar patterns were observed, with no significant differences in the rates of anemia, thrombocytopenia, neutropenia, nausea, vomiting, fatigue, abdominal pain, diarrhea, and headache between the favorable and unfavorable KELIM score groups. The necessity for dose reduction or interruption due to TEAEs, as well as discontinuation rates due to hematologic or nonhematologic TEAEs, also showed no significant differences between the two groups (Table S4).

## 4. Discussion

In this multicenter study, we compared survival outcomes and TEAEs between favorable and unfavorable KELIM in patients with newly diagnosed advanced EOC who received platinum-based chemotherapy followed by PARP inhibitors, in either upfront or interval treatment settings. Olaparib or niraparib were used as maintenance treatments in our study. Prior studies have found that both treatments exhibit efficacy and have shown comparable rates of survival [18]. In the PCS cohort, favorable KELIM was associated with improved PFS without affecting TEAEs, confirming previous findings regarding the role of KELIM in predicting PARP inhibitor efficacy [19]. Additionally, in the ICS cohort, a favorable KELIM score increased the likelihood of complete cytoreduction. The prognostic value of KELIM has been demonstrated across various treatment settings and populations, suggesting its broad applicability in EOC management, chemosensitivity prediction, and survival outcomes [13,20,21,22,23,24]. Additionally, we have proposed an optimal cutoff that maximizes the Q statistics for each threshold of the cutoff value. Although further validation is needed, it appears that the newly proposed cutoff can better predict survival outcomes in patients using PARP inhibitors. Large-scale studies are necessary for validation of this new cutoff value.

In the primary setting, the Gynecologic Cancer Intergroup (GCIG) data set, comprising 5884 individual patient data, revealed that the KELIM, evaluated using a standardized cutoff of 1.0, demonstrated an independent prognostic value for PFS and OS [22]. This analysis was conducted using a large data set before the era of PARP inhibitor maintenance treatment. Consequently, in the current clinical setting where PARP inhibitor is standard treatment used in patients with BRCA mutations, it was essential to conduct further research to evaluate the effectiveness of KELIM, taking into account the response to PARP inhibitors. In addition to the *BRCA1/2* status, the current study investigated the KELIM as a potential prognostic marker of PARP inhibitor response in advanced EOC. Categorization of patients based on KELIM scores revealed a clear distinction in PFS outcomes between those with favorable and unfavorable scores specifically in the PCS cohort, while no significant difference was observed in the ICS cohort. This suggests that KELIM, while initially promising as a marker of platinum sensitivity, may also be indicative of oncologic outcomes in the PCS cohort. The results of this study show that the KELIM score can be a marker of oncologic outcomes when combining chemotherapy and subsequent maintenance PARP inhibitor therapy. The higher proportion of patients with BRCA mutations in the current study can be attributed to the fact that, in Korea, PARP inhibitors are covered by insurance for ovarian cancer patients with BRCA mutations, while those without BRCA mutations face higher out-of-pocket costs. This socioeconomic factor could explain the high proportion of BRCA mutations observed in our study. Furthermore, niraparib usage was more prevalent in the BRCA wild-type group. The multivariate analysis, which takes into account the hazard ratio for gBRCA mutation status, suggests that the worse oncologic outcomes observed in the niraparib group are likely influenced by this distribution.

Similar results were observed in an exploratory analysis from the Velia Trial [19]. In the PCS cohort, the KELIM score was identified as a significant independent prognostic covariate (HR, 0.67; 95% CI, 0.52–0.85), alongside the treatment arm, surgery outcomes based on postoperative lesions, and HR status. However, within the ICS cohort, a larger confidence interval above 1 was noted (HR, 0.44; 95% CI, 0.19–1.00) [19]. In the current study, KELIM as a prognostic factor for PFS was not statistically significant in the ICS cohort. Previous investigation, utilizing real-world data from a cohort of 219 patients diagnosed with advanced high-grade serous ovarian cancer undergoing neoadjuvant chemotherapy, indicated poorer PFS and OS in patients with a KELIM < 1, who were likely to exhibit platinum-resistant disease, compared to those with a KELIM ≥ 1 [25]. However, the percentage of patients who received PARP inhibitor maintenance in this study was only 8.2% (18/219). Given the recognized overlap in sensitivity and resistant mechanism between platinum and PARP inhibitors [26,27,28], further analysis to verify KELIM in the ICS cohort is warranted to precisely discern and consider both platinum sensitivity and the response following PARP inhibitor treatment.

Our findings also demonstrated a significant correlation between unfavorable KELIM scores and a higher likelihood of macroscopic residual disease in the ICS cohort. Predicting cytoreductive outcomes in patients with EOC before surgery is a challenge, and there is an unmet need to improve the selection of suitable candidates for cytoreduction. There are existing measures including the Peritoneal Cancer Index (PCI) score determined by imaging and/or surgical assessment for evaluating surgical complexity [29,30], but incorporating the KELIM score as an additive model might enhance predictive accuracy for cytoreductive outcomes. Given that poorer KELIM scores suggest reduced platinum sensitivity, the impact of complete cytoreduction might be more pronounced. Nonetheless, the decision between early intervention with cytoreduction or increasing the number of neoadjuvant chemotherapy cycles remains unclear. To address this issue, future prospective studies are warranted to explore the optimal timing of ICS in correlation with KELIM scores.

The current study has limitations, including its retrospective design and the potential for selection bias. Moreover, the small number of patient populations and treatment regimens studied may restrict the generalizability of our findings. The small number of patients in each group made subgroup analysis difficult. The predictive value of KELIM in the context of other biomarkers and genetic profiles, such as BRCA mutation and HRD status, remains to be fully explored.

Despite these limitations, to our knowledge, this is the first multicenter study based on real-world experience that evaluates the association between KELIM and oncological outcomes in advanced ovarian cancer patients who received PARP inhibitors after platinum-based chemotherapy. Other studies have primarily been based on randomized controlled trials. By assessing whether favorable or unfavorable KELIM scores correlate with the incidence or severity of TEAEs, our study provides further dimensions to understanding the comprehensive implications of KELIM, not only on efficacy outcomes but also on treatment feasibility. Ongoing phase III trials are prospectively examining KELIM as a prognostic factor in recurrent EOC patients (e.g., the NIRVANA trial, which compares niraparib in the first-line setting with or without bevacizumab (NCT04734665), and the KOV-04 (FOCUS trial) and KOV-02R trial, which evaluate HIPEC for EOC including after use of PARP inhibitors (NCT05827523, NCT05316181). Future prospective studies are needed to validate our findings, ideally incorporating a broader range of PARP inhibitors including first-line settings and exploring the interplay between KELIM, genetic mutations, and other prognostic indicators.

## 5. Conclusions

A favorable KELIM score was associated with improved PFS in patients with advanced EOC undergoing primary cytoreductive surgery, highlighting its potential as a prognostic marker for the efficacy of PARP inhibitor therapy. Moreover, in patients who underwent neoadjuvant chemotherapy followed by interval cytoreductive surgery, a favorable KELIM score increased the likelihood of achieving complete cytoreduction, underscoring its utility in preoperative surgical planning.

## Figures and Tables

**Table 1:** Patients characteristics.

	PCS			ICS		
	KELIM (Favorable)	KELIM (Unfavorable)	*p* Value	KELIM (Favorable)	KELIM (Unfavorable)	*p* Value
	(*n* = 43, 28.5%)	(*n* = 108, 71.5%)		(*n* = 64, 63.4%)	(*n* = 37, 36.6%)	
KELIM score						
Median (IQR)	1.2 (1.1–1.4)	0.7 (0.6–0.9)	<0.0001	1.4 (1.3–1.7)	0.8 (0.6–0.9)	<0.0001
Age at diagnosis, years						
Median (IQR)	54 (47–61)	56.5 (49–64)	0.130	58 (52–63.1)	59 (51–65)	0.68
Histologic type			0.725			0.622
High grade serous	41 (95.4)	100 (92.6)		62 (96.9)	35 (94.6)	
Others	2 (4.7)	8 (7.4)		2 (3.1)	2 (5.4)	
FIGO stage 2014 at diagnosis			0.406			0.339
III	29 (67.4)	65 (60.2)		34 (53.1)	16 (43.2)	
IV	14 (32.6)	43 (39.8)		30 (46.9)	21 (56.8)	
*BRCA1/2* mutation status			0.906			0.935
*BRCA1/2* wild-type	18 (42.9)	47 (43.9)		23 (35.9)	13 (35.1)	
*BRCA1/2* mutation	24 (57.1)	60 (56.1)		41 (64.1)	24 (64.9)	
Maintenance treatment			0.071			0.778
Olaparib	13 (30.2)	50 (46.3)		31 (48.4)	19 (51.4)	
Niraparib	30 (69.8)	58 (53.7)		33 (51.6)	18 (48.7)	
Surgical outcome			0.700			0.037
No residual disease	22 (51.2)	59 (54.6)		38 (59.4)	14 (37.8)	
Residual disease	21 (48.8)	49 (45.4)		26 (40.6)	23 (62.2)	
Number of cycles of platinum-based chemotherapy			0.312			0.056
<6 cycles	0 (0)	4 (3.7)		7 (10.9)	2 (5.4)	
6 cycles	37 (86.1)	95 (88)		49 (76.6)	23 (62.2)	
>6 cycles	6 (14)	9 (8.3)		8 (12.5)	12 (32.4)	
Best radiological response to platinum-based chemotherapy			0.689			0.049
Complete response	40 (93)	103 (95.4)		62 (96.9)	31 (83.8)	
Partial response	3 (7)	5 (4.6)		2 (3.1)	6 (16.2)	
Serum CA-125 levels at initial diagnosis, IU/mL						
Median (IQR)	778 (289–1556)	963 (296.5–2620)	0.477	1969.5 (695–3355)	1098 (486–5000)	1

Abbreviation: PARP, poly ADP ribose polymerase; IQR, interquartile range; FIGO, International Federation of Gynecology and Obstetrics; HRD, homologous recombination deficiency; VUS, variant of uncertain significance; PV, pathogenic variant; LPV, likely pathogenic variant.

**Table 2:** Cox proportional hazards model regarding progression-free survival: (A) PCS cohort; (B) ICS cohort.

(A) PCS Cohort						
Parameter	Univariable		Multivariable (Cutoff 1.0)		Multivariable (Cutoff 0.82)	
	Hazard Ratio (95%CI)	*p* Value	Hazard Ratio (95%CI)	*p* Value	Hazard Ratio (95%CI)	*p* Value
KELIM response (cutoff 1.0)						
Favorable	1		1			
Unfavorable	3.51 (1.37–8.97)	0.009	3.03 (1.18–7.76)	0.021		
KELIM response (cutoff 0.82)						
Favorable	1				1	
Unfavorable	3.60 (1.79–7.23)	<0.001			3.64 (1.79–7.4)	<0.001
Surgical outcome						
No residual disease	1					
Residual disease	1.70 (0.9–3.21)	0.099				
Best radiological response after platinum-based chemotherapy						
CR	1					
PR	1.27 (0.39–4.15)	0.688				
gBRCA mutation						
PV/LPV	1		1		1	
wild-type/VUS	4.05 (1.85–8.86)	0.001	4.02 (1.84–8.78)	0.001	4.52 (2.06–9.94)	<0.001
Treatment						
Olaparib	1					
Niraparib	3.50 (1.55–7.92)	0.003				
**(B) ICS cohort**						
**Parameter**	**Univariable**		**Multivariable (Cutoff 1.0)**		**Multivariable (Cutoff 1.4)**	
	**Hazard Ratio (95%CI)**	***p*** **Value**	**Hazard Ratio (95%CI)**	***p*** **Value**	**Hazard ratio (95%CI)**	***p*** **Value**
KELIM response (cutoff 1.0)						
Favorable	1		1			
Unfavorable	1.42 (0.77–2.64)	0.266	1.16 (0.59–2.31)	0.665		
KELIM response (cutoff 1.4)						
Favorable	1				1	
Unfavorable	1.87 (0.89–3.92)	0.099			1.74 (0.78–3.87)	0.173
Surgical outcome						
No residual disease	1					
Residual disease	1.98 (1.05–3.75)	0.035				
Best radiological response after platinum-based chemotherapy						
CR	1		1		1	
PR	2.61 (1.15–5.91)	0.021	4.38 (1.57–12.23)	0.005	4.10 (1.56–10.79)	0.004
gBRCA mutation						
PV/LPV	1		1		1	
wild-type/VUS	2.81 (1.46–5.40)	0.002	3.66 (1.80–7.45)	<0.001	3.73 (1.83–7.60)	<0.001
Treatment						
Olaparib	1					
Niraparib	2.54 (1.30–4.99)	0.007				

## Data Availability

The data sets, statistical analysis plans, and scripts used in this study are available from the corresponding authors upon request.

## Supplementary Materials

The following supporting information can be downloaded at: https://www.mdpi.com/article/10.3390/cancers16132339/s1, Table S1. Patients’ characteristics in the Niraparib and Olaparib subgroups; Table S2. Cox proportional hazards model regarding progression-free survival in the Niraparib subgroup; Table S3. Cox proportional hazards model regarding progression-free survival in the Olaparib subgroup; Table S4. Adverse events and treatment modification.
