# Supplementary material for: Prognostic Role of CA-125 Elimination Rate Constant (KELIM) in Patients with Advanced Epithelial Ovarian Cancer Who Received PARP Inhibitors
Source: Cancers (Basel). 2024 Jun 26;16(13):2339. doi: 10.3390/cancers16132339 (PMC11240593; doi:10.3390/cancers16132339)
Supplement: Supplementary file 1 [file cancers-16-02339-s001.zip › cancers-3026722-supplementary.pdf]

Table S1. Patients' characteristics in Niraparib and Olaparib subgroups

	Niraparib			Olaparib		
	**KELIM (Favorable)**	**KELIM (Unfavorable)**	**p value**	**KELIM (Favorable)**	**KELIM (Unfavorable)**	**p value**
	**(n=63, 45.3%)**	**(n=76, 54.7%)**		**(n=44, 38.9%)**	**(n=69, 61.1%)**	
KELIM score						
Median (IQR)	1.3 (1.1-1.6)	0.7 (0.6-0.9)	**<0.001**	1.3 (1.1-1.6)	0.8 (0.6-0.9)	**<0.001**
Age at diagnosis, years						
Median (IQR)	56 (50-62.5)	60 (50.9-65)	0.266	56 (50-62)	56 (49-64)	0.895
Histologic type			0.112			0.642
High grade serous	61 (96.8)	100 (92.6)		42 (95.5)	67 (97.1)	
Others	2 (3.2)	8 (10.5)		2 (4.6)	2 (2.9)	
FIGO stage 2014 at diagnosis			0.778			0.446
III	40 (63.5)	50 (65.8)		23 (52.3)	31 (44.9)	
IV	23 (36.5)	26 (34.2)		21 (47.7)	38 (55.1)	
BRCA1/2 mutation status			0.203			0.280
BRCA1/2 wild-type	41 (66.1)	57 (76)		0 (0.0)	3 (4.4)	
BRCA1/2 mutation	21 (33.9)	18 (24)		44 (100.0)	66 (95.7)	
gBRCA mutation			0.264			0.163
wild-type/VUS	45 (77.6)	63 (85.1)		3 (7.0)	11 (15.9)	
PV/LPV	13 (22.4)	11 (14.9)		40 (93.0)	58 (84.1)	
Residual disease			0.618			**0.030**
Microscopic	28 (44.4)	37 (48.7)		32 (72.7)	36 (52.2)	
Macroscopic	35 (55.6)	39 (51.3)		12 (27.3)	33 (47.8)	
Number of cycles of platinum-based chemotherapy						
Median (range)	6 (3-9)	6 (3-11)	0.358	6 (3-11)	6 (3-15)	0.910
Best radiological response to platinum-based chemotherapy			0.756			0.080
Complete response	58 (92.1)	71 (93.4)		44 (100.0)	63 (91.3)	
Partial response	5 (7.9)	5 (6.6)		0 (0.0)	6 (8.7)	
Serum CA-125 levels at initial diagnosis, IU/ml						
Median (IQR)	1061 (524-2860)	910 (28-34900)	0.329	1243.5 (464.5-3205)	1077 (38-18000)	0.960

Table S2. Cox proportional hazards model regarding progression-free survival in Niraparib subgroup

Niraparib subgroup						
**Parameter**	**Univariable**		**Multivariable (cut-off 1)**		**Multivariable (cut-off 0.82)**	
	**Hazard ratio (95%CI)**	**p value**	**Hazard ratio (95%CI)**	**p value**	**Hazard ratio (95%CI)**	**p value**
KELIM response (cut-off 1)						
Favorable (≥1)	1		1			
Unfavorable (<1)	1.39 (0.83-2.32)	0.207	1.53 (0.89-2.65)	0.127		
KELIM response (cut off-0.82)						
Favorable (>0.82)	1				1	
Unfavorable (≤0.82)	1.93 (1.17-3.19)	**0.010**			1.92 (1.15-3.22)	**0.013**
Surgical outcome						
Microscopic	1					
Macroscopic	1.21 (0.74-1.97)	0.444				
Best radiological response after platinum-based chemotherapy						
CR	1					
PR	0.93 (0.38-2.33)	0.883				
gBRCA mutation						
PV/LPV	1		1		1	
wild-type/VUS	2.26 (1.03-4.97)	**0.043**	2.55 (1.15-5.69)	**0.022**	2.53 (1.14-5.62)	**0.023**
FIGO stage 2014 at diagnosis						
III	1		1		1	
IV	1.52 (0.93-2.47)	**0.003**	1.70 (1.01-2.87)	**0.046**	1.71 (1.02-2.89)	**0.044**

Table S3. Cox proportional hazards model regarding progression-free survival in Olaparib subgroup

Olaparib subgroup				
**Parameter**	**Univariable**		**Multivariable (cut-off 1)**	
	**Hazard ratio (95%CI)**	**p value**	**Hazard ratio (95%CI)**	**p value**
KELIM response (cut-off 1)				
Favorable (≥1)	1		1	
Unfavorable (<1)	2.48 (0.82-7.48)	0.106	3.96 (1.23-12.74)	**0.021**
Surgical outcome				
Microscopic	1			
Macroscopic	4.27 (1.68-10.84)	**0.002**		
Best radiological response after platinum-based chemotherapy				
CR	1			
PR	9.71 (3.66-25.73)	**<0.001**		
gBRCA mutation				
PV/LPV	1			
wild-type/VUS	1.36 (0.40-4.67)	0.621		
FIGO stage 2014 at diagnosis				
III	1			
IV	1.99 (0.82-4.84)	0.131		

Table S4. Adverse Events and Treatment Modification

Parameter	PCS			ICS		
	KELIM (Favorable) (n=43, 28.5%)	KELIM (Unfavorable) (n=108, 71.5%)	p value	KELIM (Favorable) (n=64, 63.4%)	KELIM (Unfavorable) (n=37, 36.6%)	p value
Anemia	23 (53.5)	70 (64.8)	0.197	40 (63.5)	23 (62.2)	0.894
Thrombocytopenia	10 (23.3)	24 (22.2)	0.891	17 (27)	7 (18.9)	0.362
Neutropenia	15 (34.9)	45 (41.7)	0.442	30 (47.6)	17 (46)	0.871
Nausea	10 (23.3)	46 (42.6)	**0.026**	20 (31.3)	11 (29.7)	0.873
Vomiting	5 (11.6)	14 (13)	0.823	5 (7.8)	6 (16.2)	0.205
Fatigue	8 (18.6)	36 (33.3)	0.072	19 (29.7)	13 (35.1)	0.571
Abdominal pain	3 (7)	12 (11.1)	0.557	10 (15.6)	4 (10.8)	0.500
Diarrhea	0 (0)	4 (3.7)	0.578	2 (3.1)	0 (0)	0.531
Headache	2 (4.7)	8 (7.4)	0.725	4 (6.3)	4 (10.8)	0.460
Dose reduction because of TEAE	22 (51.2)	60 (55.6)	0.625	32 (50)	19 (51.4)	0.896
Dose interruption because of TEAE	16 (37.2)	44 (40.7)	0.689	26 (40.6)	14 (37.8)	0.783
Discontinuation because of TEAE	2 (4.7)	6 (5.6)	1.000	3 (4.7)	2 (5.4)	1.000
Because of hematologic TEAE	2 (4.7)	4 (3.7)		2 (3.1)	1 (2.7)	
Because of non-hematologic TEAE	0 (0)	2 (1.9)		1 (1.6)	1 (2.7)	
